# Salivary Cytokines as Potential Diagnostic Biomarkers for Systemic Lupus Erythematosus Disease

**DOI:** 10.1155/2021/8847557

**Published:** 2021-03-11

**Authors:** Zeineb Zian, Assia Bouhoudan, Nadira Mourabit, Gholamreza Azizi, Mohcine Bennani Mechita

**Affiliations:** ^1^Biomedical Genomics and Oncogenetics Research Laboratory, Faculty of Sciences and Techniques of Tangier, Abdelmalek Essaadi University, Tetouan, Morocco; ^2^Faculty of Sciences of Tetouan, Abdelmalek Essaadi University, Tetouan, Morocco; ^3^Higher Institute of Nursing Professions and Technical Health of Tangier, Morocco; ^4^Non-Communicable Diseases Research Center, Alborz University of Medical Sciences, Karaj, Iran

## Abstract

Systemic lupus erythematosus (SLE) is a complex autoimmune inflammatory disease characterized by an unknown etiology and a highly variable clinical presentation. This clinical heterogeneity might be explained by dysregulation of tolerance to self and apoptotic mechanisms, overproduction of autoantibodies, and abnormal cytokine levels. Cytokine imbalance levels have been associated with disease activity and severity in SLE patients. In the last years, salivary cytokines related to SLE have gained significant attention and researchers have begun to focus on the identification of cytokines in the saliva of SLE patients using it as a diagnostic fluid for the inflammatory process underlying SLE. This review highlights and summarizes recent studies revealing the cytokines that have been identified in the saliva of individuals with SLE. Data reported and discussed in this report may provide useful additional information to better understand the mechanisms associated with the disease.

## 1. Introduction

Systemic lupus erythematosus (SLE) is an autoimmune inflammatory disease that affects many organs, including the skin, joints, central nervous system (CNS), and kidneys. It is characterized by a high female predominance (with a ratio of 9 : 1) with a common onset in young women, a large variety of clinical symptoms [1, 2], and the production of autoantibodies directed against nuclear and cytoplasmic autoantigens. Its prevalence is in the order of 178 per 100,000 habitants, with an increased incidence and severity in non-Caucasian patients [3]. Lupus nephritis (LN), with kidney inflammation, is a major cause of morbidity and mortality in up to 60% of patients with SLE and is frequently linked to a poor long-term prognosis [4]. There is a wide variation in the natural history and development of SLE among different ethnic and geographical groups. The epidemiological studies have mentioned that the disease is rare in Africa but common in African descendants around the world. Although its etiology remains not understood, SLE is known to involve a complex interaction of genetic, environmental, and immunologic factors [5]. It is known that several immune cell subsets have a significant role in the SLE pathogenesis since this is a multisystemic autoimmune disease. Thus, individuals with SLE are characterized by the dysfunction of the innate and adaptive immune systems and increased production of characteristic autoantibodies and some inflammatory mediators, including cytokines [5]. Yet, the diagnosis of SLE constitutes a challenge due to delay in the diagnostic procedure and treatment initiation, which eventually increases damage to vital organ systems and reduces treatment effectiveness.

Previous scientific evidence reported the occurrence of frequent oral manifestations in individuals with SLE [6, 7]. These implications include unspecified oral ulcers, which are reported to be more severe as the disease, xerostomia, hyposalivation [6], and increased periodontal disease (PD) [8, 9]. The PD is an infection of the tissue that supports and surrounds the tooth structure caused by microorganisms that colonize the tooth gingival interface. An association between SLE activity and periodontal status has been already demonstrated [10]. It was previously shown that saliva secretion, which is the main defensive element in the oral cavity, can be affected by some rheumatic diseases, including SLE, with subsequent negative outcomes on the teeth and mouth mucosa [6]. Yang et al. have shown that SLE increased the susceptibility of SLE patients to dental cavities by impairing saliva function and leading to imbalance in the oral microbial community [11]. Therefore, SLE patients were reported to be more vulnerable to caries infection. It was shown that 25% of SLE patients have involvement of the oral mucous membrane and lip with possible petechiae. High rates of gingivitis, cavities, PD, and missing teeth have been found in SLE patients. Hyposalivation and xerostomia can make patients with SLE predisposed to dental caries and frequent noninfectious pharyngitis and oral ulcerations [7]. Moreover, it should be noted that drugs associated with the treatment of SLE, such as corticosteroids, might cause oral candidiasis and infections [12]. On the other hand, these used drugs can influence positively the function of the exocrine gland by suppression of inflammatory glandular changes. Considering these data, salivary secretion depends on the general state of hydration, systemic diseases, and used drugs.

Some researchers have focused on the use of saliva as a diagnostic fluid for the inflammatory process underlying SLE. It was demonstrated that numerous blood biomarkers considerably correlate with respective salivary concentrations and relatively small amounts are needed for detection [13]. Thus, in the last years, some researchers have started to investigate characteristic biomarkers of SLE in saliva [14, 15]. Interestingly, recent investigations have emphasized the identification of cytokine in the saliva of patients who suffered from SLE.

To the best of our knowledge, studies focusing on detecting salivary cytokines related to SLE patients have been very scarce [9, 16–18]. In the present review, we will give an overview of the immunological biomarkers, especially the cytokine profiles that have been identified in the saliva of individuals with SLE. Data reported and discussed in this work could provide useful and additional information to better understand the mechanisms associated with the disease.

## 2. Characteristics of Saliva as a Diagnostic Fluid

In the last decades, saliva has been proved to be a reliable source for the detection of biomarkers in different local and systemic diseases [19–22] since it reflects both local and general health of the human body.

Saliva is a combination of various organic and inorganic molecules, derived from diverse salivary glands, desquamated blood and oral epithelial cells, gingival crevicular fluid (GCF), and some microorganisms [23]. By enzymatic action, lubrication, and antibacterial properties, saliva can accomplish several functions, including digestion of nutrients and protection of teeth and oral tissues [24]. Unlike other body fluids such as urine, cerebrospinal fluid (CSF), and blood, saliva has received special and remarkable attention because of its noninvasive nature and simple access and storage. It is known that saliva contains proteins rich in information about a disease process [25], is easier to handle during diagnostic procedures, and does not need specialized equipment or trained staff to obtain it. Self-collection is, therefore, possible after instruction. One of the major advantages that make saliva a potential diagnostic tool is that the specimen collection is easy and does not cause stress and discomfort for individuals, which is more acceptable to patients especially children and elderly people [23] who may accept to repeat the sample collection many times when necessary. Scientific evidence reported the use of the whole saliva (WS) instead of specific glandular secretions, for early detection and monitoring of diseases, since WS is a complex fluid that contains glandular secretions (95.6%) mixed with gingival fluid (2.4%), microorganisms (1%), and epithelial host cells (1%) [25]. Hettegger et al. have shown a high similarity of the immunological profiles in plasma and saliva for each individual by confirming that the IgG antibodies found in blood are also accessible from saliva [26]. By these results, investigators have demonstrated that the diagnostic potential of saliva is not limited to diseases localized in the oral cavity or associated structures but is extended to any IgG antibody which exists in the blood.

Salivaomics study that includes various “omics” constituents of saliva (proteome, transcriptome, micro-RNA, metabolome, and microbiome) in different fields has helped the researchers to develop new technologies to discover and quantify a wide range of salivary biomolecules. With the current technological developments, the attention to the use of saliva as an acceptable alternative to blood for use in clinical routines has been greatly increased [27]. A recent report has clearly described and explained the most important methodology techniques used for discovery, identification, and/or quantification of biomarkers in saliva, such as spectrophotometric assays (ultraviolet and visible absorption, atomic absorption, atomic emission, near-infrared, and flow injection spectrophotometric analysis), immunoassays (enzyme-linked immunoassays (EIAs), chemiluminescence immunoassays, fluoroimmunoassays, radioimmunoassays, nonlabeled immunoassays, paper based-immunoassays, and multiplex immunoassays), and “omics” techniques [23]. The choice for the technique is depending on the study objectives and the salivary biomarkers to be investigated. Several methods can be used for saliva collection, including passive drool, which is considered the gold standard [28]. These methods can also be different depending on the selected analytes and the state of patients.

In the case of SLE patients, blood collection is often difficult and causes pain and discomfort, in particular for elderly patients and those in an advanced stage of disease. Unlike urine, which is stored in the bladder for some hours before sampling, saliva samples can reflect real-time levels of biomarkers. Another characteristic of saliva is that many informative proteins can be found in human saliva, which can be used for the detection of diseases. Several blood biomarkers significantly correlate with corresponding salivary concentrations, and small amounts are required for detection. The search for new biomarkers in saliva will help in developing noninvasive diagnostic and monitoring tools for SLE patients. Recently, some investigators tested the hypothesis that salivary cytokines can represent potential biomarkers of diagnosis and monitoring of SLE patients and have measured specific cytokine concentrations in the saliva of SLE patients for their possible diagnostic utility using different analytical tests.

Although saliva has several characteristics that recommend it as a solid diagnostic specimen, it should be noted that there are some restrictions on the use of saliva for certain diseases. Some of these limitations include the types of collection methods, changes in the composition, low concentration of target analyte, and interpersonal or personal differences [23]. Moreover, in the case of SLE patients who suffered from periodontal disease [9, 16, 18], if saliva is to be collected, a thorough oral examination must be performed to rule out changes from oral inflammation. Thus, to avoid the possible bias of using saliva as a diagnostic means, the oral state of the patients should be taken into account.

## 3. Cytokines in Individuals with SLE

Cytokines are defined as small soluble mediators that can be produced by many immune cell subsets such as lymphocytes, macrophages, and dendritic cells. They are involved in the differentiation, maturation, and activation of cells and have a significant influence on the immunoinflammatory response. They include mainly interleukins (ILs), chemokines, interferons (IFNs), and members of the Tumor Necrosis Factor (TNF) family [29, 30]. Their effects are pleiotropic, which include both synergistic and antagonistic effects on other cytokines [31] and can favor the development of immune-mediated diseases. As mentioned above, cytokines have been reported to have an important role in determining the strength, nature, and duration of the immune response in the pathogenesis of SLE [32]. Cytokines such as interferons and some interleukins have been suggested to contribute significantly to the immune imbalance in SLE, while the role of TNF remains debated [33].

IL-6 is an interleukin, with pleiotropic functions, synthesized predominantly by monocytes, fibroblasts, and endothelial cells and less frequently in T and B cells. It enhances the differentiation process of naïve T cells into Th17 cells and promotes activation and/or differentiation of macrophages, neutrophils, and B and T cells that are involved in SLE [31]. Increased levels of IL-6 are found in CSF of SLE patients with psychosis [34]. In addition, a correlation between titers of anti-double-stranded DNA (dsDNA) antibodies and higher urinary levels of IL-6 has been found in patients with LN [35, 36]. SLE patients with neuropsychiatric syndromes also showed increased levels of IL-6 in the CSF [37]. Higher concentrations of IL-6 are found in serum of SLE patients compared to control individuals [17] which correlated with SLEDAI (systemic lupus erythematosus disease activity index) [38] or anti-DNA levels [39, 40]. Furthermore, increased IL-6 levels are associated with B cell hyperactivity and autoantibody production [39] and secretion of IgG anti-DNA antibodies. A significant increase was also observed in the serum levels of IL-6 in SLE patients with PD [9]. These findings suggest that IL-6 could be a link between systemic disease and local inflammation as well as a potential marker of periodontitis activity. Increased levels of IL-17 and IL-33 have been detected in SLE patients with PD on the one hand, and a positive correlation has been observed between the levels of IL-6, IL-17, and IL-33 and the changes in the oral microbiota in SLE patients on the other [9]. IL-17, commonly known as IL-17A, is a proinflammatory cytokine with multiple functions in the regulation of tissue inflammation. It amplifies the immune response in SLE by increasing the production of autoantibodies that contribute to injury of target organs [41]. Elevated levels of IL-17A have been found in SLE patients [42–44]. Furthermore, IL-17A plasma concentrations were positively correlated with SLEDAI and poor prognosis in lupus nephritis [45]. Interestingly, this cytokine is emerging as a potential therapeutic target for SLE treatment [41, 45].

Similar to IL-6, elevated levels of serum TNF-*α* have been observed in SLE patients and correlated with disease activity [39, 46]. In contrast, some studies have reported that increased TNF-*α* plasma levels did not correlate with SLE disease activity, and another report demonstrated that TNF-*α* levels were essentially higher in patients with inactive disease, suggesting a protective role for TNF-*α* in SLE [47, 48]. In fact, the role of TNF-*α* in SLE remains not clear, taking into consideration that some findings describe protective effects while other results suggest harmful effects on the disease. These discrepancies may be explained by differences in patient characteristics and used methods of identification of this cytokine. A positive correlation has also been found between increased levels of type I IFN and disease activity in some cross-sectional studies, while other ones could not establish correlations [49–51]. The type I IFN family involves at least 13 IFN-*α* subtypes and IFN-*β*, IFN-*ω*, IFN-*κ*, IFN-*ε*, and IFN-*α* which is the most studied among all members. Higher serum levels of type I interferon-regulated chemokines have previously been suggested as biomarkers for disease activity [52]. This was later proven by associations between increased transcript levels of these chemokines with disease activity and organ damage [53, 54]. Patients with SLE frequently exhibited enhanced levels of IFN-*α* serum, which correlated with the production of anti-dsDNA antibodies and disease activity, complement activation, and circulating IL-10 [55]. This suggested that elevated serum IFN-*α* activity might be a heritable risk factor for SLE [56]. Higher levels of IFN-*γ* have also been reported associated with disease activity in SLE patients. Increased levels of IL-18 in SLE sera were strongly correlated with active disease (SLEDAI > 8) [57]. B-lymphocyte stimulator (BLyS), also known as B cell activating factor (BAFF), which is a crucial cytokine for the survival of most B cells beyond the transitional 1 stage, was also found with higher levels in up to 50% of SLE patients [58].

On the other hand, several cytokines (IL-18, IFN-*γ*, IL-12p70, IL-6, and IL-17A) were higher in the serum of patients with LN class IV in comparison to healthy control [59]. IL-6 also mediates local inflammation in LN [60]. Furthermore, the serum cytokines IL-18, IL-17A, and IFN-*γ* have been markedly expressed in LN-IV patient glomeruli and interstitial tissue than other classes of LN. These findings suggest that the early detection of these cytokines could contribute to the prediction of the active form of LN-IV.  Table 1 summarizes the most cytokines studied in patients with SLE in different types of fluids.

## 4. Salivary Cytokines in Individuals with SLE

In the search for a noninvasive diagnostic method, some serum inflammatory cytokines of SLE were investigated, for their diagnostic potential, in the saliva of patients suffering from SLE (Table 2). Although it is important to use saliva as a source of biomarkers in SLE, little information, from our knowledge, is available regarding the cytokine profile in the saliva of SLE patients so far. This was confirmed by some studies investigating salivary cytokines in individuals with this autoimmune disease. The Stanescu et al. [17] study was the first to use saliva for monitoring IL-6 levels and the inflammatory status in SLE. In their work, higher salivary levels of IL-6 have been reported in SLE patients. They also found a positive correlation between salivary and serum levels of IL-6, which indicates that salivary IL-6 could be a reliable marker to evaluate the inflammation process in SLE. Monocyte chemoattractant protein-1 (MCP-1) salivary concentrations were also found higher in SLE patients compared to the control individuals, and no statistical correlation has been found between saliva and serum levels [17]. A recent study has shown significantly increased salivary levels of IL-6 and IL-17A in SLE patients with PD compared to controls with PD [18]. This high concentration of salivary IL-17A of SLE patients with PD could suggest periodontium as the SLE target structure. In contrast, previous results reported no significant difference in IL-17 salivary concentrations of SLE patients with and without PD [16]. On the other hand, concentrations of IL-6 and IL-1*β* have been found lower in saliva of SLE patients with PD when compared to healthy controls with PD [16]. In fact, many studies have shown that PD and SLE have a similar immunological profile, including altered levels of inflammatory cytokines in the saliva of SLE patients, and a high prevalence of PD in SLE patients [9, 16, 18]. In a meta-analysis, the risk of PD in SLE patients was increased with a risk ratio of 1.76 [77]. Interestingly, a positive correlation has been found between high salivary levels of IL-1*β* and IL-4 and periodontal status in SLE patients. Thus, these salivary cytokines have been suggested to be promising biomarkers to predict the destruction of PD in patients with SLE [16]. A previous study conducted by Jung et al. [78] has shown a positive correlation between salivary IL-1*β* levels and disease duration in SLE patients. A multivariate model showed association of increased IL-33 salivary concentration with high SLE activity. Moreover, concentrations of salivary IL-33 were higher in SLE subjects with PD compared to SLE without PD, which indicates that periodontitis increased the salivary concentration of IL-33 in SLE patients [18]. Corrêa et al. [9] investigated previously the relationship between bacteria and local inflammation by assessing cytokines in the saliva of SLE patients with and without PD. Changes in the oral microbiota were linked to high local inflammation, as exhibited by increased levels of IL-6, IL-17, and IL-33 in SLE patients with PD. Furthermore, no significant differences in cytokine concentrations were observed in the control group with PD when compared to the control group without PD. Taking into consideration these findings, the association between SLE and PD could be indicated by the increased salivary levels of cytokine in SLE patients. Furthermore, such changes in cytokine levels could have an important role in disseminating inflammation in SLE.

In line with these data, saliva continues to be a gold standard biofluid to monitor the activity and progression of SLE disease with or without PD. However, when interpreting the levels of salivary cytokines in these patients, it is necessary to take into consideration that cytokines present in saliva can be produced by GCF, the oral gingival cells, and cell groups [79] on the one hand and the WS contains inhibitors such as mucin-like proteins and other proteases that can reduce until 75% the concentration of cytokines when revealed using Luminex [80], on the other.

## 5. Future Perspectives and Concluding Remarks

Taking into consideration these findings, which indicated that salivary cytokine concentrations were increased in SLE patients, saliva could be considered having the potential to be an important diagnostic fluid for this disease. However, the potentiality of saliva as a good diagnostic tool can have some limitations. The detection technologies should be more sensitive to detect a low number of analytes that are present in saliva. Another limitation is the lack of understanding of saliva biology and variations in saliva composition, especially the lack of knowledge related to the correlation between biomolecules in blood with saliva and the circadian variations of biomolecules in saliva [19]. One other important limitation that should be mentioned is the possible bias of using saliva as a diagnostic means due to the possible alteration of oral health (periodontal health) of the patients, which could modify the results. SLE disease could be better understood by technological and analytical advances in proteomics. Using saliva instead of blood in routine diagnostics will have potential benefits because of its noninvasive nature, safety, and easy sampling with information related to body health. It is important to be aware and conscious of the interest of developing and validating a new based saliva diagnostic test for the most reproducible cytokine markers of SLE, which can be used as an inexpensive screening tool and could be more efficient for identifying and monitoring patients in several stages of the disease. The development of specific and standardized analytical tools should take into consideration the cost-effectiveness, time, and ease of use. Reducing the number of false-negative and false-positive test result outcomes is one of the main characteristics of future tests because these will have detrimental effects on patient diagnosis and subsequently the therapy.

Although salivary cytokines in SLE patients have been assessed in different studies, these investigations remained very scarce and were only of a small sample. Therefore, more studies with larger sample size are needed to confirm the findings documented in the literature. Salivary cytokine study needs more attention, development, and validation, in particular regarding which cytokine panel correlates with disease onset and progression in order to determine the cytokine profile with high diagnostic sensitivity and specificity. Thus, early detection of SLE through the identification of cytokines present in saliva might lead to early intervention, which may, therefore, reduce the morbidity and mortality associated with this medical condition. Besides, investigations monitoring the changes of the salivary level of these biomarkers after SLE treatment are recommended. Further research on potential salivary prognostic cytokines in SLE patients is still required. Salivary cytokines could be helpful indicators of SLE severity and disease progression and might serve as valuable and reliable diagnostics for early detection of SLE in the near future.

## Figures and Tables

**Table 1:** Cytokine profiles identified in SLE patients.

Study	Subjects (*n*)	Sample types	Cytokines measured	Analytical methods	Findings
Selvaraja et al. [61]	64 SLE patients (17 with and 47 without LN)/15 HC	Plasma	IL-25, IL-4, IL-5, IL-6, IL-9, IL-10, and IL-13	ELISA and multiplex cytokine assay	(i) Significantly increased levels of IL-9, IL-10, and IL-25 in SLE patients with and without LN (ii) Significantly reduced levels of IL-5 and IL-6 (iii) No significant difference was observed with IL-13 (iv) Level of IL-4 was undetectable (v) IL-25, IL-9, and IL-10 were positively correlated with the disease severity score
Torell et al. [62]	310 SLE patients/310 HC	Serum	IFN-*γ*, IL-6, IL-8, IL-10, IL-15, IL-16, CXCL10, MCP-1, MIP-1*α*, MIP-1*β*, TNF-*α*	Mesoscale discovery multiplex analysis	(i) Increased serum levels of IL-10, TNF-*α*, IL-15, MIP-1*α*, CXCL10, MCP-1, IL-6, IFN-*γ*, MIP-1*β*, IL-16, IL-8 in SLE patients
Reynolds et al. [63]	96 SLE patients/13 HC	Serum	CXCL13, BLyS, IL-18, PTX3, CXCL10, IL-17, IFN-*α*, IL-10, IL-21, MCP-1	High-sensitivity bead-based multiplex ELISA	(i) Serum levels of BLyS, IL-17, IL-18, CXCL10, and PTX3 were significantly higher in SLE patients with active disease
Guimarães et al. [64]	200 SLE patients/196 HC	Plasma	IL-6, IL-12, IL-17, IFN-*γ*, IL-10, IL-4, TNF-*α*, IL-6, IL-1*β*	ELISA	(i) Significantly higher levels of IL-6, IL-12, IL-17, IFN-*γ*, and IL-10 in SLE (ii) Lower levels of IL-4 in SLE (iii) No significant differences in (TNF-*α*+IL-6+IL-1*β*) profile
Arora et al. [65]	40 SLE patients/40 HC	Serum	IL-4, IFN-*γ*, IL-10, and TNF-*α*	ELISA	(i) In HC: levels of IFN-*γ* were highest, followed by TNF-*α*, IL-10, and IL-4 (ii) In SLE patients: levels of TNF-*α* were highest, followed by IFN-*γ*, IL-10, and IL-4 (iii) IL-10 and IL-4 correlated negatively, and IFN-*γ* and TNF-*α* correlated positively with the SLEDAI scores
Shah et al. [66]	25 SLE patients/26 HC	Serum	IL-1*β*, IL-6, IL-10, TGF-*β*, IL-21, and IL-23	ELISA and multiplex cytokine assay	(i) Higher levels of IL-6 in SLE than HC (ii) No differences in levels of IL-1*β*, IL-21, IL-23, and TGF-*β* between SLE and HC
Li et al. [67]	56 SLE patients/30 HC	Serum	IL-27	ELISA	(i) Significantly lower serum level of IL-27 in SLE
Yang et al. [68]	50 SLE patients/15 HC	Serum	IL-17A and IFN-*γ*	ELISA	(i) Higher levels of IL-17A in SLE patients with active disease
Petri et al. [69]	245 SLE patients	Serum	BLyS	ELISA	(i) Elevated BLyS serum levels were in SLE patients and correlate with disease activity
Chun et al. [38]	166 SLE patients/167 DC (90 with RA and 77 with AS) and 40 HC	Serum	IL-2, IL-6, IL-10, IL-12, IFN-*γ*	ELISA	(i) SLE patients had higher IL-6, IL-10, IL-12, and IFN-*γ* levels, but lower IL-2, than HC (ii) Levels of IL-6 and IL-10 were significantly higher in active SLE patients and correlated with the SLE activity index (SLEDAI)
Niewold et al. [56]	266 SLE patients and their 405 healthy relatives/105 healthy unrelated	Serum	IFN-*α*	ELISA	(i) Serum level of IFN-*α* in both patients and healthy relatives was higher as compared to healthy unrelated individuals
Baranda et al. [70]	18 SLE patients/14 HC	Serum	IL-15, IFN-*γ*, and TNF-*α*	ELISA	(i) Increased serum levels of IL-15 in SLE patients
Capper et al. [71]	74 SLE patients/13 HC	Serum	IL-10, IL-12, IL-1RA	ELISA	(i) Higher levels of circulating IL-10 and IL-1RA in SLE patients (ii) Significantly higher levels of circulating IL-12 in SLE patients
Gómez et al. [48]	52 SLE patients/25 HC	Serum	TNF-*α*, IFN-*γ*, IL-12p70, IL-10, IL-4	ELISA	(i) Levels of IFN-*γ*, TNF-*α*, and IL-12 were significantly higher in SLE patients
Calvani et al. [72]	133 SLE patients with and without LN/44 HC	Serum	IL-18, IFN-*γ*, IL-4	ELISA	(i) Higher serum IL-18 in SLE (ii) High IFN-*γ* with low IL-4 expression in SLE patients with LN (iii) A positive correlation between serum IL-18 and IFN-*γ* levels
McCarthy et al. [73]	45 SLE patients/20 HC	Serum	IFN-*γ*, TNF-*α*, IL-1*β*, IL-12p70, IL-10	ELISA	(i) Elevated levels of IL-1*β*, TNF-*α*, IFN-*γ*, and IL-10 in SLE (ii) Increased levels of IL-12p70 coincided with increased levels of IL-10 in SLE patients (iii) A significant relationship between IL-10, TNF-*α*, and IL-1 *β* and disease activity
Min et al. [74]	40 SLE patients (17 with LN and 23 without LN)/80 HC	Serum	IFN-*γ*, IL-12, IL-4, and IL-10	ELISA	(i) Significantly higher levels of serum IFN-*γ*, IL-12, IL-4, and IL-10 in SLE patients than HC (ii) Significantly lower levels of serum IL-12 and IFN-*γ* in patients with LN compared to those without (iii) Significantly higher levels of serum IL-10 in patients with LN compared to those without
Gröndal et al. [40]	52 SLE patients/29 HC	Serum	IL-6, IL-10	ELISA	(i) Serum levels of IL-10 and IL-6 were increased in SLE patients (ii) Serum IL-10 levels correlated with the titer of anti-dsDNA antibodies in the patients
Tsai et al. [75]	27 LN patients (15 with active LN and 12 with inactive)/17 HC	Urine	IL-6, IL-8, IL-2R, and *β*2M	ELISA	(i) The urinary excretions of IL-6 and IL-8 were significantly higher in patients with active LN than in those with inactive LN and HC (ii) The urinary *β*2M excretion in LN patients was significantly higher than that in HC
Wong et al. [76]	36 SLE patients/18 HC	Serum	IL-17, IL-12, IL-18, and IL-4	ELISA	(i) Increased serum levels of IL-12, IL-17, IL-18, and IL-4 in SLE patients

AS: ankylosing spondylitis; BD: Behcet's disease; BLyS: B-lymphocyte stimulator; *β*2M: beta 2-microglobulin; CNS: central nervous system; CSF: cerebrospinal fluid; DC: disease control; ELISA: enzyme-linked immunosorbent assay; HC: healthy controls; IL: interleukins; IL-1RA: interleukin 1 receptor antagonist; INF: interferon; LN: lupus nephritis; MCP: monocyte chemoattractant protein; MIP: macrophage inflammatory protein; NS: nephrotic syndrome; PMR: polymyalgia rheumatica; PTX3: pentraxine 3; RA: rheumatoid arthritis; SLE: systemic lupus erythematosus; SS: Sjogren's syndrome; TNF: tumor necrosis factor; TP: thrombocytopenia.

**Table 2:** Salivary cytokine profiles in SLE patients.

Study	Subjects (*n*)	Salivary cytokines measured	Analytical methods	Findings
Mendonça et al. [18]	70 SLE patients (with or without PD)/70 HC (with or without PD)	IL-33, IL-17A, IL-6	ELISA/cytometric bead array	(i) Significantly higher salivary levels of IL-6 and IL-17A in SLE-PD compared to HC-PD (ii) Lower levels of IL-33 in SLE-non-PD compared to HC-non-PD (iii) The PD significantly increased the levels of IL-6, IL-17A, and IL-33 in saliva of SLE patients compared to SLE-non-PD (iv) Periodontitis seemed to affect SLE individuals earlier
Stanescu et al. [17]	24 SLE patients/5 HC	IL-6, leptin, MCP-1, and PAI-1	Stochastic sensors	(i) Significantly higher salivary levels of IL-6 in SLE patients (ii) Salivary IL-6 levels highly correlated with the serum IL-6 levels in SLE patients
Corrêa et al. [9]	52 SLE patients (with or without PD)/52 HC (with or without PD)	IL-6, IL-17, IL-33, TNF-*α*, and IFN-*γ*	(i) Flow cytometer (ii) ELISA	(i) Significantly increased saliva levels of IL-6, IL-17, and IL-33 in SLE-PD compared to HC-PD (ii) Changes in the oral microbiota were linked to increased local inflammation, as demonstrated by higher concentrations of IL-6, IL-17, and IL-33 in SLE-PD patients
Marques et al. [16]	60 SLE patients (30 with and 30 without PD)/54 HC (27 with and 27 without PD)	IFN-*γ*, IL-10, IL-17, IL-1*β*, IL-4, and IL-6	Automatic analyzer MAGPIX system	(i) Higher salivary levels of IFN-*γ*, IL-10, IL-17, IL-1*β*, IL-6, and IL-4 in SLE even in the absence of PD (ii) Higher salivary levels of IL-1*β* and IL-6 in HC-PD compared to SLE-PD and SLE-non-PD (iii) IL-1*β* and IL-4 salivary levels were positively correlated with periodontal status indicating their potential as markers of the amount and extent of periodontal damage in SLE patients

ELISA: enzyme-linked immunosorbent assay; HC: healthy controls; IL: interleukins; IL-1RA: interleukin 1 receptor antagonist; INF: interferon; MCP: monocyte chemoattractant protein; PD: periodontal disease; SLE: systemic lupus erythematosus; TNF: tumor necrosis factor.

## References

[1] Petri M. (2008). Sex hormones and systemic lupus erythematosus. *Lupus*.

[2] Zian Z., Maamar M., Aouni M. E. (2018). Immunological and clinical characteristics of systemic lupus erythematosus: a series from Morocco. *BioMed Research International*.

[3] Stojan G., Petri M. (2018). Epidemiology of systemic lupus erythematosus: an update. *Current Opinion in Rheumatology*.

[4] Saxena R., Mahajan T., Mohan C. (2011). Lupus nephritis: current update. *Arthritis Research & Therapy*.

[5] Kaul A., Gordon C., Crow M. K. (2016). Systemic lupus erythematosus. *Nature Reviews. Disease Primers*.

[6] Jensen J. L., Bergem H. O., Gilboe I.-M., Husby G., Axéll T. (1999). Oral and ocular sicca symptoms and findings are prevalent in systemic lupus erythematosus. *Journal of Oral Pathology & Medicine*.

[7] Hammoudeh M., Al-Momani A., Sarakbi H., Chandra P., Hammoudeh S. (2018). Oral manifestations of systemic lupus erythematosus patients in Qatar: a pilot study. *International Journal of Rheumatology*.

[8] Kobayashi T., Ito S., Yamamoto K. (2003). Risk of periodontitis in systemic lupus erythematosus is associated with Fc*γ* receptor polymorphisms. *Journal of Periodontology*.

[9] Corrêa J. D., Calderaro D. C., Ferreira G. A. (2017). Subgingival microbiota dysbiosis in systemic lupus erythematosus: association with periodontal status. *Microbiome*.

[10] Wu Y.-C., Ning L., Tu Y.-K. (2018). Salivary biomarker combination prediction model for the diagnosis of periodontitis in a Taiwanese population. *Journal of the Formosan Medical Association*.

[11] Yang L., Wang J., Xiao Y. (2018). Saliva dysfunction and oral microbial changes among systemic lupus erythematosus patients with dental caries. *BioMed Research International*.

[12] Atherley G., Glasgow K., Taylor L., Fraser K. (2012). College of Dental Hygienists of Ontario Advisory Lupus. http://www.cdho.org/Advisories/CDHO_Advisory_Lupus.pdf.

[13] Yoshizawa J. M., Schafer C. A., Schafer J. J., Farrell J. J., Paster B. J., Wong D. T. W. (2013). Salivary biomarkers: toward future clinical and diagnostic utilities. *Clinical Microbiology Reviews*.

[14] Zaieni S. H., Derakhshan Z., Sariri R. (2015). Alternations of salivary antioxidant enzymes in systemic lupus erythematosus. *Lupus*.

[15] Franceschi L. D., Bosello S., Scambi C. (2011). Proteome analysis of biological fluids from autoimmune-rheumatological disorders. *PROTEOMICS - Clinical Applications*.

[16] Marques C. P. C., Victor E. C., Franco M. M. (2016). Salivary levels of inflammatory cytokines and their association to periodontal disease in systemic lupus erythematosus patients. A case-control study. *Cytokine*.

[17] Stanescu I.-I., Calenic B., Dima A. (2018). Salivary biomarkers of inflammation in systemic lupus *erythematosus*. *Anz.*.

[18] Mendonça S. M. S., Corrêa J. D., Souza A. F. (2019). Immunological signatures in saliva of systemic lupus erythematosus patients: influence of periodontal condition. *Clinical and Experimental Rheumatology*.

[19] Pfaffe T., Cooper-White J., Beyerlein P., Kostner K., Punyadeera C. (2011). Diagnostic potential of saliva: current state and future applications. *Clinical Chemistry*.

[20] Helenius L. M. J., Meurman J. H., Helenius I. (2005). Oral and salivary parameters in patients with rheumatic diseases. *Acta Odontologica Scandinavica*.

[21] Miricescu D., Totan A., Calenic B. (2014). Salivary biomarkers: relationship between oxidative stress and alveolar bone loss in chronic periodontitis. *Acta Odontologica Scandinavica*.

[22] Zian Z., Bakkach J., Barakat A., Ghailani Nourouti N., Bennani Mechita M. (2018). Salivary biomarkers in systemic sclerosis disease. *BioMed Research International*.

[23] Tvarijonaviciute A., Martínez-Subiela S., López-Jornet P., Lamy E., Tvarijonaviciute A., Martínez-Subiela S., López-Jornet P., Lamy E. (2020). The future of saliva as an analytical sample. *Saliva in Health and Disease: The Present and Future of a Unique Sample for Diagnosis*.

[24] Mandel I. D. (1987). The functions of saliva. *Journal of Dental Research*.

[25] Esteves C. V., Campos W. G., Souza M. M., Lourenço S. V., Siqueira W. L., Lemos-Júnior C. A. (2019). Diagnostic potential of saliva proteome analysis: a review and guide to clinical practice. *Brazilian Oral Research*.

[26] Hettegger P., Huber J., Paßecker K. (2019). High similarity of IgG antibody profiles in blood and saliva opens opportunities for saliva based serology. *PLoS One*.

[27] Kaczor-Urbanowicz K. E., Martin Carreras-Presas C., Aro K., Tu M., Garcia-Godoy F., Wong D. T. (2016). Saliva diagnostics – current views and directions. *Exp. Biologie et Médecine*.

[28] Khurshid Z., Zohaib S., Najeeb S., Zafar M. S., Slowey P. D., Almas K. (2016). Human saliva collection devices for proteomics: an update. *International Journal of Molecular Sciences*.

[29] Dinarello C. A. (2007). Historical insights into cytokines. *European Journal of Immunology*.

[30] Aringer M., Smolen J. S. (2005). Cytokine expression in lupus kidneys. *Lupus*.

[31] Jacob N., Stohl W. (2011). Cytokine disturbances in systemic lupus erythematosus. *Arthritis Research & Therapy*.

[32] Ohl K., Tenbrock K. Inflammatory cytokines in systemic lupus erythematosus. https://www.hindawi.com/journals/bmri/2011/432595/.

[33] Lourenço E. V., Cava A. L. (2009). Cytokines in systemic lupus erythematosus. *Current Molecular Medicine*.

[34] Hirohata S., Kanai Y., Mitsuo A., Tokano Y., Hashimoto H. (2009). Accuracy of cerebrospinal fluid IL-6 testing for diagnosis of lupus psychosis. A multicenter retrospective study. *Clinical Rheumatology*.

[35] Peterson E., Robertson A. D., Emlen W. (1996). Serum and urinary interleukin-6 in systemic lupus erythematosus. *Lupus*.

[36] Iwano M., Dohi K., Hirata E. (1993). Urinary levels of IL-6 in patients with active lupus nephritis. *Clinical Nephrology*.

[37] Alcocer-Varela J., Aleman-Hoey D., Alarcon-Segovia D. (1992). Interleukin-1 and interleukin-6 activities are increased in the cerebrospinal fluid of patients with CNS lupus erythematosus and correlate with local late T-cell activation markers. *Lupus*.

[38] Chun H.-Y., Chung J.-W., Kim H.-A. (2007). Cytokine IL-6 and IL-10 as biomarkers in systemic lupus erythematosus. *Journal of Clinical Immunology*.

[39] Tackey E., Lipsky P. E., Illei G. G. (2016). Rationale for interleukin-6 blockade in systemic lupus erythematosus. *Lupus*.

[40] Gröndal G., Gunnarsson I., Rönnelid J., Rogberg S., Klareskog L., Lundberg I. (2000). Cytokine production, serum levels and disease activity in systemic lupus erythematosus. *Clinical and Experimental Rheumatology*.

[41] Li D., Guo B., Wu H., Tan L., Chang C., Lu Q. (2015). Interleukin-17 in systemic lupus erythematosus: a comprehensive review. *Autoimmunity*.

[42] Krebs C. F., Schmidt T., Riedel J.-H., Panzer U. (2017). T helper type 17 cells in immune-mediated glomerular disease. *Nature Reviews. Nephrology*.

[43] Zhao X.-F., Pan H.-F., Yuan H. (2010). Increased serum interleukin 17 in patients with systemic lupus erythematosus. *Molecular Biology Reports*.

[44] Vincent F. B., Northcott M., Hoi A., Mackay F., Morand E. F. (2013). Clinical associations of serum interleukin-17 in systemic lupus erythematosus. *Arthritis Research & Therapy*.

[45] La Paglia G. M. C., Leone M. C., Lepri G. (2017). One year in review 2017: systemic lupus erythematosus. *Clinical and Experimental Rheumatology*.

[46] Heinrich P. C., Castell J. V., Andus T. (1990). Interleukin-6 and the acute phase response. *The Biochemical Journal*.

[47] Zhu L.-J., Landolt-Marticorena C., Li T. (2010). Altered expression of TNF-*α* signaling pathway proteins in systemic lupus erythematosus. *The Journal of Rheumatology*.

[48] Gómez D., Correa P. A., Gómez L. M., Cadena J., Molina J. F., Anaya J.-M. (2004). Th1/Th2 cytokines in patients with systemic lupus erythematosus: is tumor necrosis factor *α* protective?. *Seminars in Arthritis and Rheumatism*.

[49] Dall’Era M. C. (2005). Type I interferon correlates with serological and clinical manifestations of SLE. *Annals of the Rheumatic Diseases*.

[50] Landolt-Marticorena C., Bonventi G., Lubovich A. (2009). Lack of association between the interferon-*α* signature and longitudinal changes in disease activity in systemic lupus erythematosus. *Annals of the Rheumatic Diseases*.

[51] Kirou K. A., Lee C., George S., Louca K., Peterson M. G. E., Crow M. K. (2005). Activation of the interferon-*α* pathway identifies a subgroup of systemic lupus erythematosus patients with distinct serologic features and active disease. *Arthritis and Rheumatism*.

[52] Bauer J. W., Baechler E. C., Petri M. (2006). Elevated serum levels of interferon-regulated chemokines are biomarkers for active human systemic lupus erythematosus. *PLoS Medicine*.

[53] Fu Q., Chen X., Cui H. (2008). Association of elevated transcript levels of interferon-inducible chemokines with disease activity and organ damage in systemic lupus erythematosus patients. *Arthritis Research & Therapy*.

[54] Bauer J. W., Petri M., Batliwalla F. M. (2009). Interferon-regulated chemokines as biomarkers of systemic lupus erythematosus disease activity: a validation study. *Arthritis and Rheumatism*.

[55] Bengtsson A. A., Sturfelt G., Truedsson L., Blomberg J., Alm G., Vallin H. (2016). Activation of type I interferon system in systemic lupus erythematosus correlates with disease activity but not with antiretroviral antibodies. *Lupus*.

[56] Niewold T., Hua J., Lehman T., Harley J., Crow M. (2007). High serum IFN- _*α*_ activity is a heritable risk factor for systemic lupus erythematosus. *Genes and Immunity*.

[57] Bossu P., Neumann D., del Giudice E. (2011). IL-18 cDNA vaccination protects mice from spontaneous lupus-like autoimmune disease. *Proceedings of the National Academy of Sciences*.

[58] Stohl W., Looney R. J. (2006). B cell depletion therapy in systemic rheumatic diseases: different strokes for different folks?. *Clinical Immunology*.

[59] Sigdel K. R., Duan L., Wang Y. (2016). Serum cytokines Th1, Th2, and Th17 expression profiling in active lupus nephritis-IV: from a southern Chinese Han population. *Mediators of Inflammation*.

[60] Esposito P., Balletta M. M., Procino A., Postiglione L., Memoli B. (2009). Interleukin-6 release from peripheral mononuclear cells is associated to disease activity and treatment response in patients with lupus nephritis. *Lupus*.

[61] Selvaraja M., Abdullah M., Arip M., Chin V. K., Shah A., Nordin S. A. (2019). Elevated interleukin-25 and its association to Th2 cytokines in systemic lupus erythematosus with lupus nephritis. *PLoS One*.

[62] Torell F., Eketjäll S., Idborg H. (2019). Cytokine profiles in autoantibody defined subgroups of systemic lupus erythematosus. *Journal of Proteome Research*.

[63] Reynolds J. A., McCarthy E. M., Haque S. (2018). Cytokine profiling in active and quiescent SLE reveals distinct patient subpopulations. *Arthritis Research & Therapy*.

[64] Guimarães P. M., Scavuzzi B. M., Stadtlober N. P. (2017). Cytokines in systemic lupus erythematosus: far beyond Th1/Th2 dualism lupus: cytokine profiles. *Immunology and Cell Biology*.

[65] Arora V., Verma J., Marwah V., Kumar A., Anand D., Das N. (2012). Cytokine imbalance in systemic lupus erythematosus: a study on northern Indian subjects. *Lupus*.

[66] Shah K., Lee W.-W., Lee S.-H. (2010). Dysregulated balance of Th17 and Th1 cells in systemic lupus erythematosus. *Arthritis Research & Therapy*.

[67] Li T.-T., Zhang T., Chen G.-M. (2010). Low level of serum interleukin 27 in patients with systemic lupus erythematosus. *Journal of Investigative. Medicine*.

[68] Yang J., Chu Y., Yang X. (2009). Th17 and natural Treg cell population dynamics in systemic lupus erythematosus. *Arthritis and Rheumatism*.

[69] Petri M., Stohl W., Chatham W. (2008). Association of plasma B lymphocyte stimulator levels and disease activity in systemic lupus erythematosus. *Arthritis and Rheumatism*.

[70] Baranda L., de la Fuente H., Layseca-Espinosa E. (2005). IL-15 and IL-15R in leucocytes from patients with systemic lupus erythematosus. *Rheumatology (Oxford, England)*.

[71] Capper E. R., Maskill J. K., Gordon C., Blakemore A. I. F. (2004). Interleukin (IL)-10, IL-1ra and IL-12 profiles in active and quiescent systemic lupus erythematosus: could longitudinal studies reveal patient subgroups of differing pathology?. *Clinical and Experimental Immunology*.

[72] CALVANI N., RICHARDS H. B., TUCCI M., PANNARALE G., SILVESTRIS F. (2004). Up-regulation of IL-18 and predominance of a Th1 immune response is a hallmark of lupus nephritis. *Clinical and Experimental Immunology*.

[73] McCarthy E. M., Smith S., Lee R. Z. (2014). The association of cytokines with disease activity and damage scores in systemic lupus erythematosus patients. *Rheumatology (Oxford, England)*.

[74] Min D.-J., Cho M.-L., Cho C.-S. (2009). Decreased production of interleukin-12 and interferon-? is associated with renal involvement in systemic lupus erythematosus. *Scandinavian Journal of Rheumatology*.

[75] Tsai C. Y., Wu T. H., Yu C. L., Lu J. Y., Tsai Y. Y. (2000). Increased excretions of beta2-microglobulin, IL-6, and IL-8 and decreased excretion of Tamm-Horsfall glycoprotein in urine of patients with active lupus nephritis. *Nephron*.

[76] Wong C. K., Ho C. Y., Li E. K., Lam C. W. (2000). Elevation of proinflammatory cytokine (IL-18, IL-17, IL-12) and Th2 cytokine (IL-4) concentrations in patients with systemic lupus erythematosus. *Lupus*.

[77] Rutter-Locher Z., Smith T. O., Giles I., Sofat N. (2017). Association between systemic lupus erythematosus and periodontitis: a systematic review and meta-analysis. *Frontiers in Immunology*.

[78] Jung J.-Y., Nam J.-Y., Kim H.-A., Suh C.-H. (2015). Elevated salivary alpha-amylase level, association between depression and disease activity, and stress as a predictor of disease flare in systemic lupus erythematosus: a prospective case–control study. *Medicine (Baltimore)*.

[79] Sandros J., Karlsson C., Lappin D. F., Madianos P. N., Kinane D. F., Papapanou P. N. (2000). Cytokine responses of oral epithelial cells to Porphyromonas gingivalis infection. *Journal of Dental Research*.

[80] Wozniak K. L., Arribas A., Leigh J. E., Fidel P. L. (2002). Inhibitory effects of whole and parotid saliva on immunomodulators. *Oral Microbiology and Immunology*.

